# Quality evaluation based on color grading: quality discrimination of the Chinese medicine Corni Fructus by an E-eye

**DOI:** 10.1038/s41598-019-53210-5

**Published:** 2019-11-18

**Authors:** Cui YongXia, Liu RuiXin, Lin ZhaoZhou, Chen PengJu, Wang LiLi, Wang YanLi, Chen SuiQing

**Affiliations:** 1 Henan University of Chinese Medicine, Zhengzhou, 450008 China; 2 Department of Pharmacy, The First Affiliated Hospital of Henan University of CM, Zhengzhou, 450000 China; 3 Beijing Institute of Chinese Materia Medica, Beijing, 100035 China; 4 Present Address: Beijing Hospital of Traditional Chinese Medicine, Capital Medical University, Beijing, 100010 P.R. China

**Keywords:** Scientific data, Software

## Abstract

‘Quality evaluation based on color grading’ is one of the features used in Chinese medicine discrimination. In order to assess the feasibility of electronic eye (E-eye) in implementing ‘quality evaluation based on color grading’, the present study applied an IRIS VA400 E-eye to test 58 batches of Corni Fructus samples. Their optical data were acquired and combined with their corresponding classes. A total of four quality discrimination models were produced according to discrimination analysis (DA), least squares support vector machine (LS-SVM), partial least squares-discrimination analysis (PLS-DA), and principal component analysis-discrimination analysis (PCA-DA). The accuracy rate of the aforementioned 4 cross evaluation models were 86.21%, 89.66%, 81.03% and 91.38%, respectively. Therefore, the PCA-DA method was used to build the final discrimination model for classifying Corni Fructus or discriminating its quality.

## Introduction

The commodity specification and grade of Chinese medicinal materials is used as a way to harmonize the chemical information from these specimens in a standard uniform template that can be used in the Chinese medicinal market. The comprehensive and unique way to evaluate the quality of Chinese medicine can offer great significance in ensuring the quality and clinical medication safety of Chinese medical decoction^1^. At present, Chinese medicinal materials are graded largely according to their origin and appearance. One of the parameters used is color, which is considered as an important indicator of Chinese medicinal quality. From ancient times, Chinese medicinal practice has been based on a practical experience to identify the authenticity and quality of the Chinese medicine by color. This is usually called “quality evaluation based on color grading”. The determination of the specification and grade by their appearance requires practical experience and lacks quantification standards. In practice, different analyzers may draw different conclusions. Along with the related technological development, a few novel methods and techniques have been extensively applied in grading Chinese medicine, such as DNA barcode identification^2–4^, X- ray diffraction^5^, and chromatographic analysis^6–8^. These methods are accurate, reproducible and versatile in terms of their identification, whereas they also have drawbacks, such as complicated pre-processing and slow identification rates. Electronic eye (e-eye) is a bionics-based detection instrument for recognizing and analyzing visual information, which can analyze the overall color of a sample. It is usually reported in the classification of food and crops as well as in the evaluation of the product quality^9–11^. It is further applied in certain studies of drug screening^12,13^ and in quality control of film coating^14,15^. However, it is rarely reported in Chinese medicine quality evaluation.

Corni Fructus is the pulp of the ripe fruit of *Cornus officinalis* Sieb. et Zucc. (Cornaceae). The fruit is collected during late autumn and early winter when turning red, and baked with gentle heat or transiently soaked in boiling water, prior to removal of the kernel. The final step of the methodology involves drying. Corni Fructus has the biological activity of ‘tonifying the liver and kidney, promoting astriction and stemming desertion’^16^. In modern pharmacological studies, Corni Fructus indicated significant biological activities with regard to immunoregulation, cardiotonic effects, inhibition of shock and arrhythmias, hypoglycemia, antioxidation and anti-aging^17–20^. A survey demonstrated that Corni Fructus could change its color to purplish-black from its original bright red color if stored improperly. This in turn reduced the purchasing value and medicinal quality of the herb, thereby influencing its production and clinical usage. Currently, the specification and grade of Corni Fructus is primarily determined by medicinal farmers and practitioners with considerable experience, who can make decisions based on the medicinal material origin, appearance, and manufacturing methods. This strategy hardly meets the current market requirements and it is not sufficient to guide the rapid development of the Corni Fructus decoction market. Moreover, inadequate experience of the medicinal practitioners may be one of the contributing factors that can hinder the accurate recognition of the Corni Fructus. Based on this evidence, the aim of the present study was to produce a discrimination model for the Corni Fructus specification and grade, and to build a more scientific and objective quality evaluation system by the concept of ‘quality evaluation based on color grading’ in order to rapidly and precisely identify the specification and grade of medicinal materials.

## Results

### Grading standards and results

The standards of the 4 classes were customed for the Corni Fructus samples tested in the present study (Table 1). According to these standards, 58 samples were graded (Table 2).

**Table 1:** Grading standards for Corni Fructus samples.

Class	Appearance	Ratio of fruit kernel over the whole fruit
1	Bright red; crinkled, shiny; dark red area ≤10%	≤1%
2	Dark red; crinkled, shiny; reddish brown area ≤15%	≤3%
3	Reddish brown; crinkled, shiny; purplish black area ≤15%	≤3%
4	Purplish black; crinkled	≤3%

Note: all four classes shared the same requirements of “fruit flesh was irregular, flaky or cystic; sour and astringent; no impurity, moth eaten or mildew”.

**Table 2:** Class of 58 Corni Fructus samples.

No.	class	Place of origin	Place of purchase
101	1	Xixia, Henan province	Taiping Town, Xixia, Henan province
102	1		Xiping Town, Xixia, Henan province
103	1		Xixia, Henan province
104	1		Taiping Town, Xixia, Henan province
105	1		Sangping Town, Xixia, Henan province
106	1	Luanchuan, Henan province	Jiaohe Town, Luanchuan, Henan province
107	1	Xixia, Henan province	China (Bozhou) Chinese medicinal material trading center
201	2	Xixia, Henan province	Xixia, Henan province
202	2	Luanchuan, Henan province	Baitu Town, Luanchuan, Henan province
203	2	Xixia, Henan province	Genfang Village, Xixia, Henan province
204	2	Luanchuan, Henan province	Qiuba Town, Luanchuan, Henan province
205	2	Henan province	Hebei Anguodongfang Medicine Tower
206	2	Shaanxi province	Hebei Anguodongfang Medicine Tower
207	2	Henan province	Hebei Anguodongfang Medicine Tower
208	2	Neixiang, Henan province	Zhongjing Wanxi Pharmaceutical company
209	2	Henan province	Hebei Anguodongfang Medicine Tower
210	2	Song county, Henan province	Checun Town, Song County, Henan province
211	2	Nanzhao, Henan province	Badi Village, Nanzhao, Henan province
212	2	Nanzhao, Henan province	Wallnut tree Village, Nanzhao, Henan province
213	2	Nanzhao, Henan province	Tianqiao Village, Nanzhao, Henan province
214	2	Neixiang, Henan province	Zhongjing Wanxi Pharmaceutical company
215	2	Luanchuan, Henan province	Jiaohe Town, Luanchuan, Henan province
216	2	Luanchuan, Henan province	Taowan Town, Luanchuan, Henan province
217	2	Luanchuan, Henan province	Heyu Town, Luanchuan, Henan province
218	2	Henan province	China (Bozhou) Chinese medicinal material trading center
219	2	Neixiang, Henan province	Zhongjing Wanxi Pharmaceutical company
220	2	Xixia, Henan province	Xixia, Henan province
221	2	Luanchuan, Henan province	Baitu Town, Luanchuan, Henan province
222	2	Neixiang, Henan province	Zhongjing Wanxi Pharmaceutical company
223	2	Neixiang, Henan province	Zhongjing Wanxi Pharmaceutical company
224	2	Luanchuan, Henan province	China (Bozhou) Chinese medicinal material trading center
225	2	Shaanxi province	Hebei Anguodongfang Medicine Tower
226	2	Shaanxi province	Hebei Anguodongfang Medicine Tower
227	2	Shaanxi province	Hebei Anguodongfang Medicine Tower
228	2	Shaanxi province	Hebei Anguodongfang Medicine Tower
229	2	Shaanxi province	Hebei Anguodongfang Medicine Tower
230	2	Shaanxi province	Hebei Anguodongfang Medicine Tower
301	3	Xixia, Henan province	Xixia, Henan province
302	3	Zhejiang province	China (Bozhou) Chinese medicinal material trading center
303	3	Henan province	China (Bozhou) Chinese medicinal material trading center
304	3	NA	NA
305	3	Zhejiang province	China (Bozhou) Chinese medicinal material trading center
306	3	Xixia, Henan province	Xixia, Henan province
307	3	Xixia, Henan province	Xixia, Henan province
401	4	NA	NA
402	4	Xixia, Henan province	Xixia, Henan province
403	4	Xixia, Henan province	Xixia, Henan province
404	4	Xixia, Henan province	Xixia, Henan province
405	4	Henan province	China (Bozhou) Chinese medicinal material trading center
406	4	Henan province	China (Bozhou) Chinese medicinal material trading center
407	4	Xixia, Henan province	Xixia, Henan province
408	4	Taibai County, Henan province	Taochuan Town, Taibai County, Henan province
409	4	Xixia, Henan province	Xixia, Henan province
410	4	Xixia, Henan province	Xixia, Henan province
411	4	Xixia, Henan province	Xixia, Henan province
412	4	Xixia, Henan province	Xixia, Henan province
413	4	Xixia, Henan province	Xixia, Henan province
414	4	Xixia, Henan province	Xixia, Henan province

Note: “NA” referred to “Not Available”.

### E-eye test

**Figure 1:**
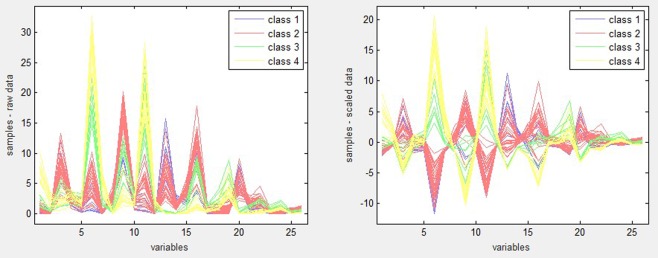
The landscape of Corni Fructus samples depicted with 26 color numbers.

Optical information of 58 Corni Fructus samples was normalized into 45 color numbers, of which 26 shared color numbers were treated as variables. Following a search in the AlphaSoft data processing software, 26 color numbers were shown to represent specific colors, as described in Table 3, and the color numbers 1075–2423 respectively represent 1–26 variables. Following grading, the landscape of 58 samples was depicted by 26 color numbers and displayed in the line chart of Fig. 1. In the optical information of the 4 classes of Corni Fructus, those samples in the same class presented a consistent trend. It indicates that the level of manual division in the early stage is better, the difference between the levels is relatively obvious, and the difference within the level is not obvious. At the same time, from the point of view of a color number variable, some levels have a negative correlation with their values. For example, from the sixth variable (1348 color number), the smaller the value, the higher the level. Samples from grade 1 to grade 4 were distributed from low to high.

**Table 3:** 26 color numbers representing specific colors.

Color no.	Color description	Color no.	Color description
1075	dark grayish reddish brown	1861	dark purplish red
1076	very dark purple	1876	moderate brown
1331	dark reddish brown	1877	dark grayish red
1332	dark purplish red	1878	dark purplish red
1347	dark grayish yellowish brown	1893	grayish yellowish brown
1348	dark reddish gray	1894	dark reddish gray
1349	dark grayish purple	2133	grayish red
1603	moderate brown	2149	moderate brown
1604	dark grayish red	2150	grayish red
1605	dark purplish red	2166	grayish yellowish brown
1621	dark reddish gray	2167	grayish red
1622	dark grayish purple	2422	light brown
1860	dark red	2423	grayish red

### Construction of multiple discrimination models and results of cross validation DA discrimination model

**Figure 2:**
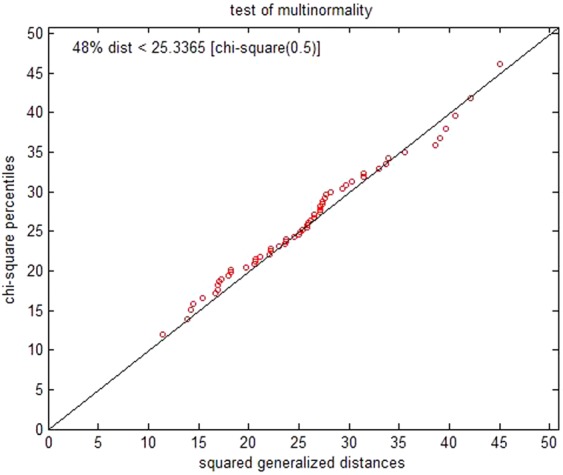
Multinormality test of 58 samples.

Figure 2 displayed the diagram of the multinormality test for 58 samples. The squared generalized distance was the horizontal coordinate and the percentiles with the Chi-squared value were the longitudinal coordinate. The data indicated that 1) the samples were nearly distributed by a linear pattern, 2) approximately 48% of the distance was estimated to less than the test value corresponding to Chi-squared percentiles of 0.5 and 26 degrees of freedom in the Chi-squared distribution quantile table. This indicated normal distribution of the data. Table 4 displayed the discriminated results of accurately or inappropriately classified samples, and the cross validation detected the accurate discrimination rate of the constructed DA model that was estimated to 86.21%.

**Table 4:** Discrimination of the DA model detected by leave-one-out cross validation.

real/predicted	class 1	class 2	class 3	class 4	not assigned	accuracy
class 1	6	1	0	0	0	86.21%
class 2	4	26	0	0	0	
class 3	0	0	6	1	0	
class 4	0	0	2	12	0	

### LS-SVM discrimination model

Leave-one-out cross validation was adopted to optimize the kernel function type of LS-SVM and other parameters. Screening was performed in linear kernel function, polynomial kernel function, multi layer perceptron kernel function, and radial basis function (RBF). RBF kernel function was ultimately selected due to its optimal effect (kernel function parameter value 1, kernel function parameter value 2); simplex and gridsearch optimization functions were subjected to screening, and simplex optimization function was ultimately selected. The accurate discrimination rate of constructed LS-SVM model was estimated to 89.66% by leave-one-out cross validation, and Table 5 displayed the sample discrimination results. The accuracy rate of the building models constructed for the overall samples was 98.28%, and the RBF kernel function parameters gamma and sig2 were 0.355 and 17.245, respectively.

**Table 5:** Discrimination of the LS-SVM model by leave-one-out cross validation.

real/predicted	class 1	class 2	class 3	class 4	not assigned	accuracy
class 1	5	2	0	0	0	89.66%
class 2	2	28	0	0	0	
class 3	0	0	6	1	0	
class 4	0	0	1	13	0	

### PLS-DA discrimination model

**Figure 3:**
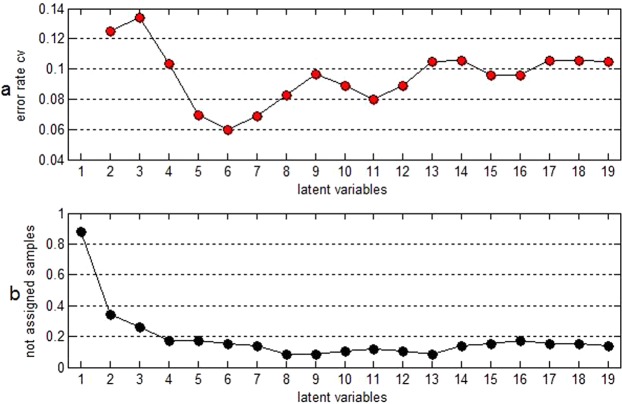
(**a**) Chart of “latent variables-error rate”; (**b**) chart of “latent variables-percentage of not assigned samples”.

**Figure 4:**
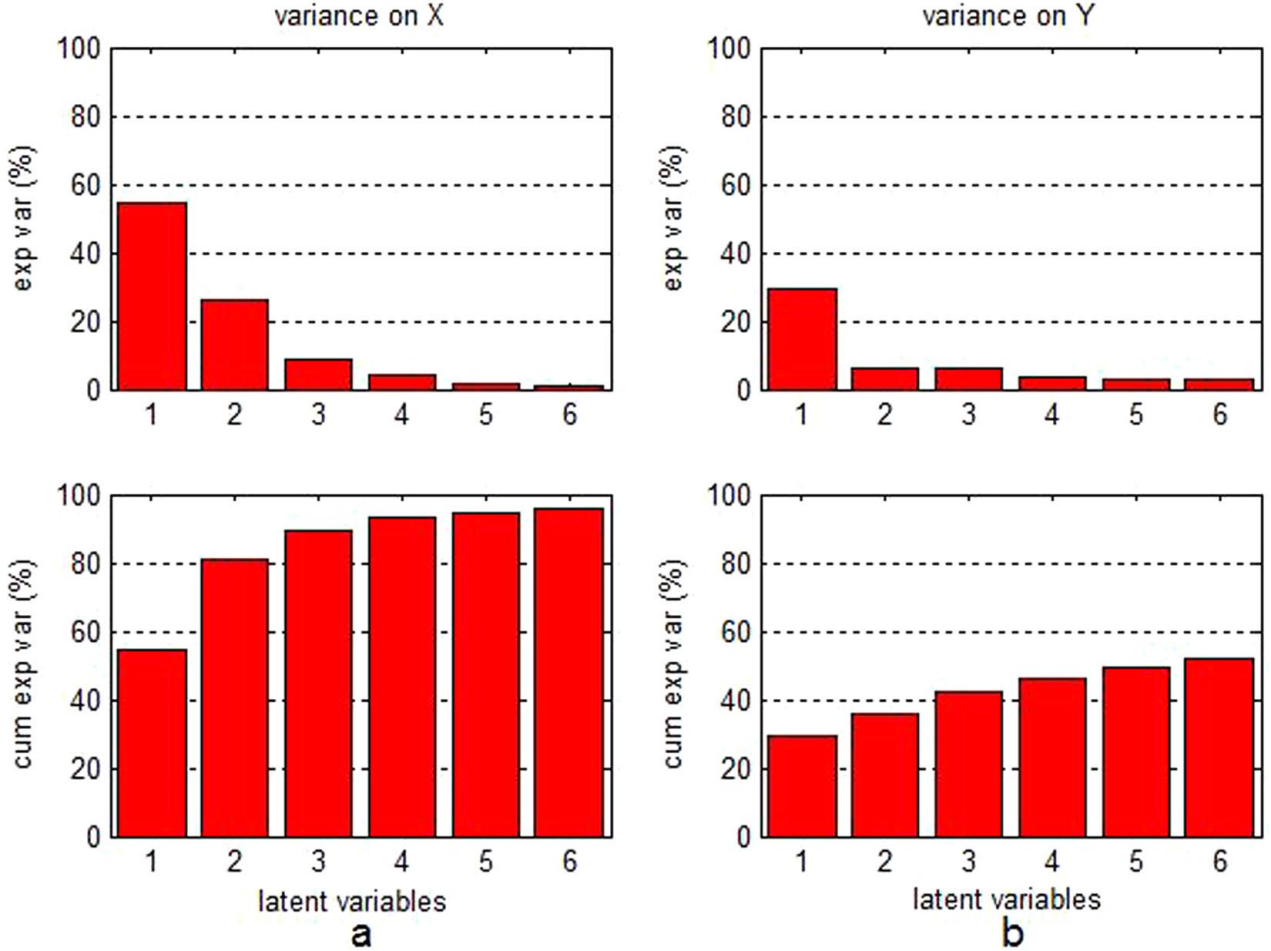
Variance contribution percentage of latent variables on independent variables (**a**) and dependent variables (**b**).

The “Latent variables-error rate” and the “latent variables-percentage of not assigned samples” charts in Fig. 3 exhibited the model performance that reached the optimal value, when the number of latent variables was 6. The cumulative variance contribution percentage in Fig. 4 indicated the selected 6 latent variables that could interpret over 99% of differential information of independent variables and approximately 50% of differential information of dependent variables.

**Figure 5:**
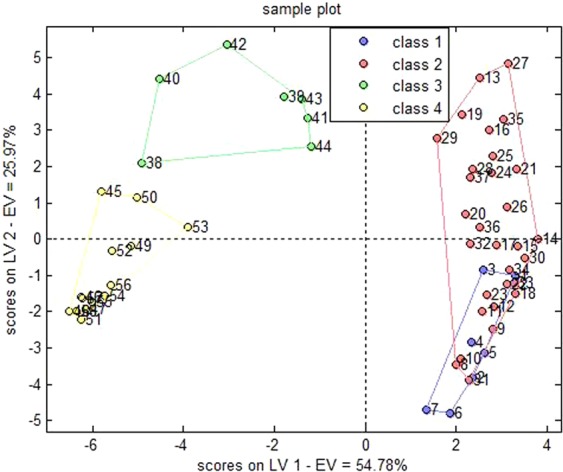
Chart of scores on latent variables in PLS-DA model.

Table 6 displayed the sample discrimination results. The accurate discrimination rate of the constructed PLS-DA model was estimated at 81.03% by leave-one-out cross validation. Figure 5 displayed the chart of scores on the latent variables. The data described in Table 6 and Fig. 5 demonstrated a small portion of overlapped class 1 & 2 samples, which could be separated from class 3 & 4 samples. However, 9 samples could not be classified by the model, namely those with color numbers of 101, 102, 202, 204, 205, 224, 301, 401, and 409. The accurate discrimination rate of this model could reach 95.91% if these 9 samples were excluded.

**Table 6:** Discrimination results of the PLS-DA model that were detected with leave-one-out cross evaluation.

real/predicted	class 1	class 2	class 3	class 4	not assigned	accuracy
class 1	4	1	0	0	2	81.03%
class 2	1	25	0	0	4	
class 3	0	0	6	0	1	
class 4	0	0	0	12	2

### PCA-DA discrimination model

**Figure 6:**
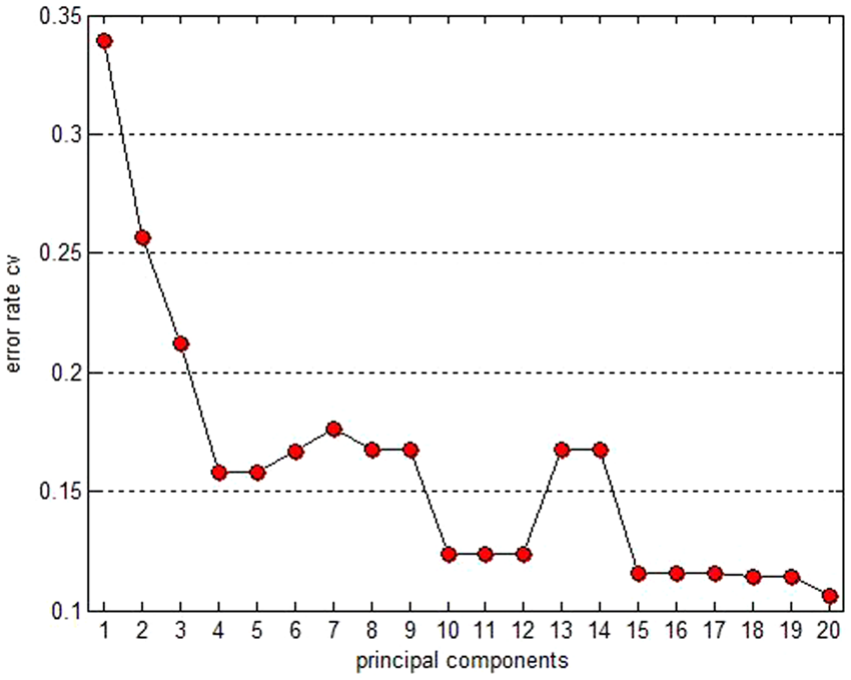
The relation of principal components-error rate.

**Figure 7:**
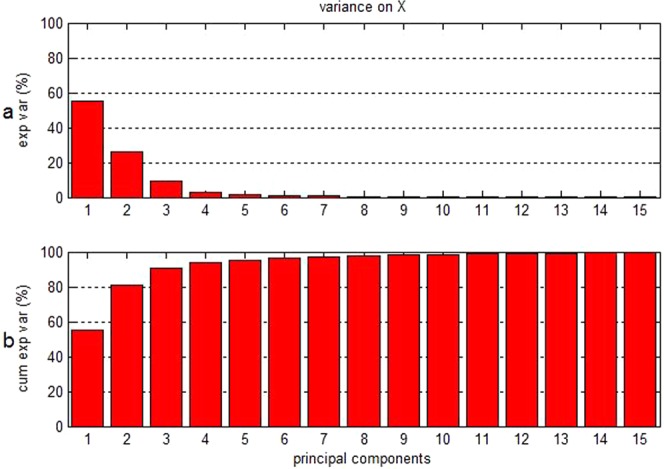
(**a**) Variance contribution percentage of individual principle components on variables; (**b**) cumulative variance contribution percentage of principle components on variables.

Principle components were selected to generate a chart of “principal components-error rate”, as shown in Fig. 6. Cross validation was conducted to compare the models constructed with 4, 10, 15, and 20 principle components, resulting in the selection of 15 principle components for model construction. The interpretation of the variation information reached over 99% in sum, and the majority of the information from the original variables could be interpreted (Fig. 7).

**Figure 8:**
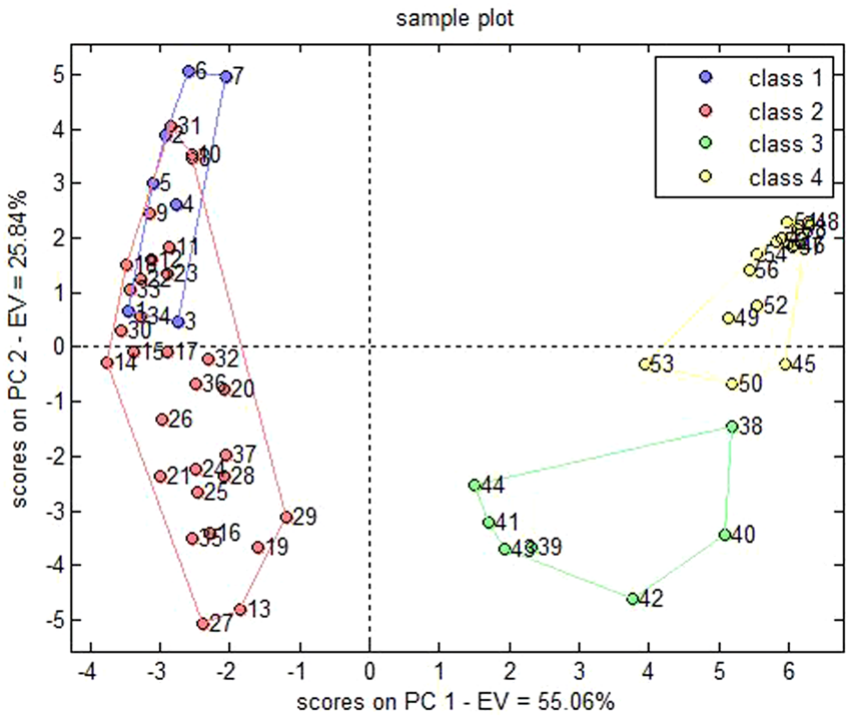
Chart of scores on principle components in the PCA-DA model.

Table 7 displayed discrimination results of correctly or incorrectly classified samples. The accurate discrimination rate of the constructed PCA-DA model was estimated to 91.38% by leave-one-out cross validation, and the chart of scores on model principle components was produced (Fig. 8). The data indicated that a limited number of samples of 4 classes overlapped, presenting high discrimination power, without unassigned samples (Table 7, Fig. 8).

**Table 7:** Discrimination results of the PCA-DA model that were detected with the leave-one-out cross validation.

real/predicted	class 1	class 2	class 3	class 4	not assigned	accuracy
class 1	6	1	0	0	0	91.38%
class 2	1	29	0	0	0	
class 3	0	0	6	1	0	
class 4	0	0	2	12	0

### The optimal full-sample discrimination model

**Figure 9:**
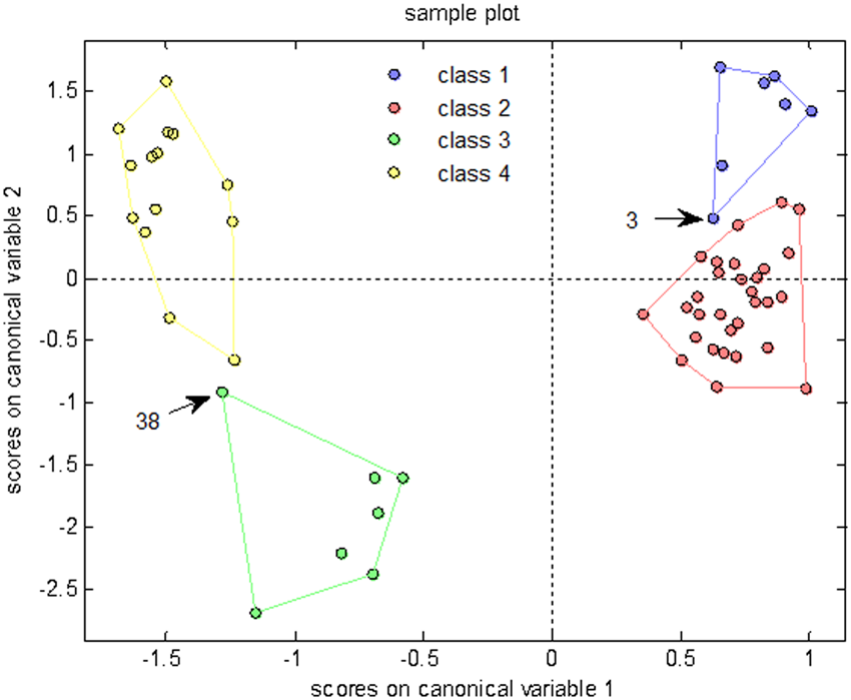
Chart of scores on canonical variables of the PCA-DA model.

In the model evaluation, the PCA-DA cross validation indicated the highest discrimination accuracy and exhibited no unassigned samples. Therefore, PCA-DA was selected as the mathematical statistical method to build the optimal model. Ultimately, the constructed PCA-DA full-sample discrimination model exhibited an accuracy rate of 98.28%, and the discrimination results were displayed in Table 8. Figure 9 exhibited the chart of scores on the model canonical variables. The data in Table 8 and Fig. 9 demonstrated that in the samples of 4 classes, only 1 sample (No. 3) was inappropriately discriminated, indicating high discriminating power, without unassigned samples. Furthermore, No. 3 and No. 38 samples at the margin belonged to the type of samples that were predisposed to inaccurate discrimination (Fig. 9).

**Table 8:** Discrimination results of PCA-DA full-sample model.

real/predicted	class 1	class 2	class 3	class 4	not assigned	accuracy
class 1	6	1	0	0	0	98.28%
class 2	0	30	0	0	0	
class 3	0	0	7	0	0	
class 4	0	0	0	14	0

## Discussion

### Contribution of individual e-eye color numbers (variables) on discrimination models

**Figure 10:**
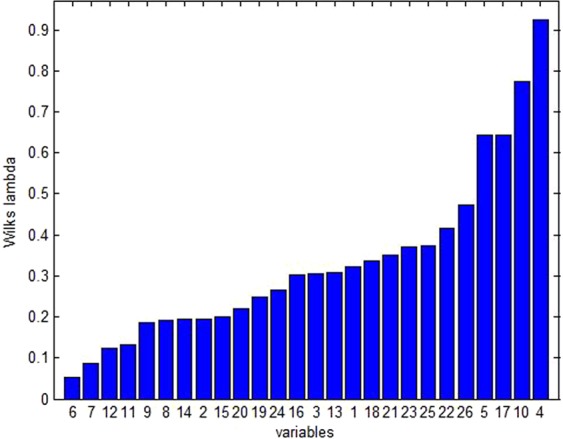
Variation information determined by 26 variables.

The bar chart in Fig. 10 displayed variation information (Wilks lamda values) that was defined by 26 variables. The lower the number of Wilks lamda values, the higher the variation information determined by the variables. The sequential order of former 6 color numbers (variables) was the following: No. 6 variable (color number 1348, described as dark reddish gray), No. 7 variable (color number 1349, described as dark grayish purple), No. 12 variable (color number 1622, described as dark grayish purple), No. 11 variable (color number 1621, described as dark reddish gray), No. 9 variable (color number 1604, described as dark grayish red), No. 8 variable (color number 1603, described as moderate brown).

**Figure 11:**
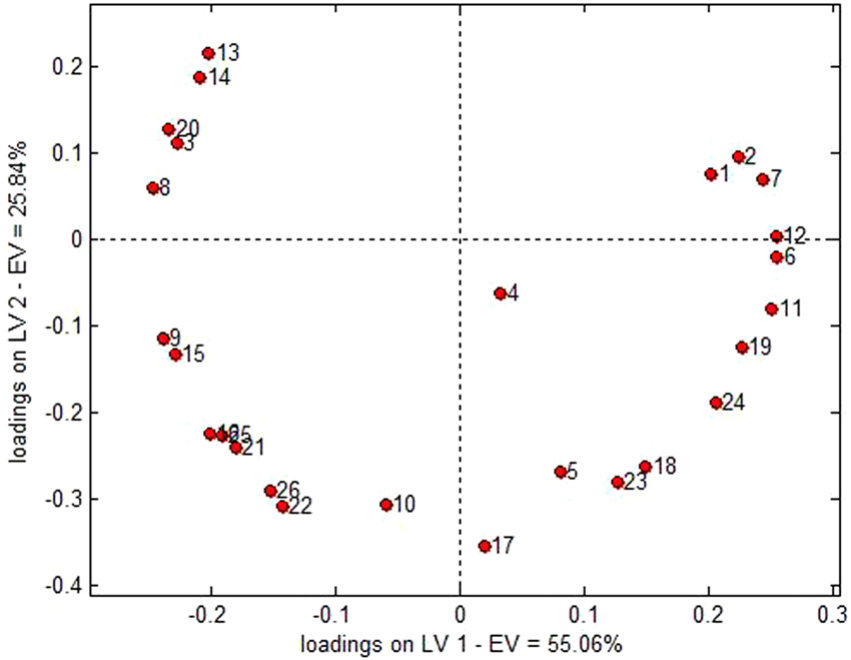
Loading diagram of latent variables in the PCA-DA full-sample model.

Figure 11 depicted the loading diagram of latent variables by 26 variables. With the exception of the No. 4 variable that was near the base point, the remaining 25 variables were generally dispersed, indicating that all 25 variables carried significant variation information. The most influential variables for the latent variable 1 were No. 6/12/11/7 (positive correlation) and No. 8/9 (reverse correlation), that were consistent with the Wilks lamda value analysis. The most influential variable for the latent variable 2 was No. 17 (reverse correlation) and No. 13 (positive correlation); No. 4 variable was located near the base point, indicating limited contribution of its feature fluctuation on the discrimination of the samples.

## Analysis on Unassigned Samples and Inappropriately Classified Samples

### Reference significance of unassigned samples

In the present study, only the PLS-DA model exhibited 9 unassigned samples. This result could be attributed to the discrimination mechanism of the PLS-DA model. These 9 samples failed to meet the standards required for the 4 classes. This indicated that the PLS-DA method was inappropriate for building discrimination models of the 58 Corni Fructus samples tested in the present study.

The unassigned samples further indicated that PLS-DA exhibited more stringent requirements over the other 3 modeling methods. The adequate size of the training set ensured that unassigned samples exhibited no significant influence on the 4 discrimination models. However, the limited size of the training set resulted in unassigned samples that could affect the model margin when recruited into other discrimination models. These discrepancies may be attributed to offset values that could be corrected by enlarging the training set and inspecting the sample processing.

In other words, following exclusion of these 9 samples, the new PLS-DA discrimination model was built, of which the accurate discrimination rate was increased to 97.92% by cross validation in the absence of unassigned samples. The data indicated that the PLS-DA model could probably act as a way to screen the sample library.

### Analysis on inappropriately classified samples

**Figure 12:**
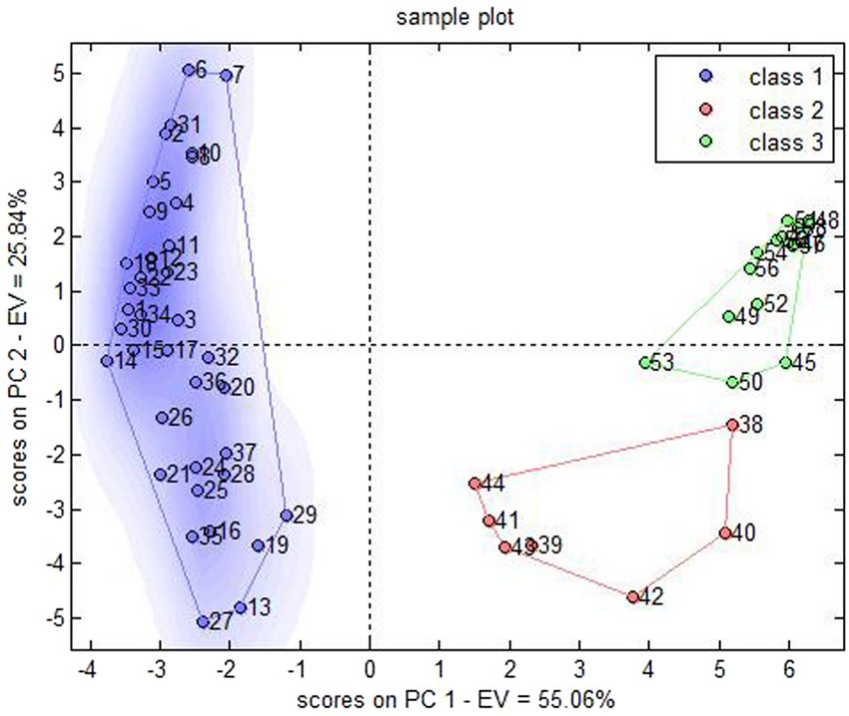
Chart of scores on principle components in the PCA-DA model in the presence of only 3 classes.

The data from Tables IV–VII demonstrated 16 sets of discrimination for 4 classes that were implemented by 4 modeling methods. All deviations in the classification were in the range of one grade or lower; all incorrect classifications were between classes 1 and 2 or between classes 3 and 4. The results indicated that the artificial standards for the specification and class were customed for Corni Fructus and were consecutive and rational. However, the proximity between classes 1 and 2 and/or classes 3 and 4 was closer. The data further reflected that the differential information acquired by the e-eye sensor in the different classes of Corni Fructus was complete. If needed, the rapid and gross classification could combine class 1 with class 2, and the accurate discrimination rate of the cross validation could subsequently reach 94.83%, 96.55%, 94.83%, and 98.28% for DA, LS-SVM, PLS-DA, and PCA-DA, respectively. Figure 12 depicted the chart of scores on the principle components when the PCA-DA exhibited the highest discrimination accuracy and there were only 3 classes; If class 3 and class 4 continue to merge, the accurate discrimination rate of the cross validation could rise to 100% in the 3 models, with the exception of the LS-SVM model that could reach 98.28%.

### E-eye sensor analysis

In the present study, the mechanism of the e-eye sensor referred to sample discrimination by acquiring sample color information. The traditional “eye view” used for discrimination contained appearance information that was defined by “color” and “shape” (such as “quality evaluation based on shape grading”). The E-eye sensor can provide valuable biological information and is an important tool that can be used for further development.

## Conclusions

The present study adopted 4 different mathematical statistics to construct models for analysis of Corni Fructus information collected by e-eye. The accurate discrimination rate of the constructed cross validation discrimination models was as follows: PCA-DA > LS-SVM > DA > PLS-DA. The models were all within the range of 81–92%, and optimal classification power was achieved. Following screening, the accuracy rate was 91.38% and 98.28% for the selected PCA-DA cross validation and the full-sample models, indicating optimal predictive results in the classification of the quality of the Corni Fructus. For the Chinese medicinal materials, of which the quality was closely related to color, such as Corni Fructus, the use of the e-eye sensor in this model provided a new conception to evaluate the quality or to implement grading that was different from the previous methods used by sensory organs or chemical constituents. The current study validated the feasibility of the e-eye in the ‘quality evaluation based on color grading’ for this type of medicinal materials.

## Materials and Methods

### Instruments and materials

E-eye experiments were conducted with an IRIS VA400 e-eye (Alpha M.O.S, Toulouse, France). The instrument was equipped with 16 million color industrial camera and 6700 K color temperature D65 international standard light source.

A total of 58 samples of Corni Fructus were from the primary places of origin and medicinal material markets distributed in Henan, Zhejiang, Shanxi, and Shaanxi provinces of China. All samples were identified by Prof. Suiqing CHEN at Henan University of CM as the pulp of the ripe fruit of Cornus officinalis Sieb. et Zucc. (Cornaceae). Furthermore, in accordance with the “Chinese Pharmacopoeia I” (edition 2015), the components Monoside (C_17_H_26_O_11_) and Loganin (C_17_H_26_O_10_) were identified in the water-soluble extracts of all individual samples examined.

Discrimination models were built with MATLAB R2010b (Mathwork Inc.) and the classification toolbox for MATLAB-version 5.0^21^. The LS-SVM classification model was built with the LS-SVMlab Toolbox^22^.

### Sample grading

Sample grading was conducted by 6 specialists from Henan University with expertise in Chinese medicinal quality standards or authenticity analysis. They were familiar with all processes of Corni Fructus planting, manufacturing and circulation. In addition, the grading standard for Corni Fructus was established according to the “*Chinese Pharmacopoeia*” (edition 2015) and Corni Fructus commodity specification requirements stated in “*commodity specification and grade standards for 76 Chinese medicinal materials*”, and in accordance to relevant literature and the market circulation guidelines. A total of 6 specialists were trained according to the standard guidelines prior to the grading of 58 samples. The class of individual samples was determined by the principle of no less than 2/3 proportion. The samples with less than 2/3 specialists supporting a certain grade were excluded from this study.

### E-eye detection

The E-eye was switched on and checked for light stability (green light) prior to the initiation of the detection. The calibration was performed with a 24-color correction board, and the background was eliminated by simultaneous turning on the upper and lower backlights with a 5 mm aperture. Following calibration, the samples of the unified thickness were placed evenly on the watch glass, followed by image acquisition for each sample. Triplicate images were acquired for each sample at exchanged positions. Raw images were processed with the software equipped with a RGB/L*a*b*/HSV multicolor processing system. The images were split into 4096 colors for analysis and similar colors were subjected to normalizing, thus generating the result of color distribution.

### Construction of multiple discrimination models

Discrimination models were constructed respectively by four distinct methods including discrimination analysis (DA), least squares-support vector machine (LS-SVM), partial least squares-discrimination analysis (PLS-DA), and principal component analysis-discrimination analysis (PCA-DA).

### Construction of DA discrimination model

Linear discrimination analysis aimed to identify a projection direction in the high dimension space, which could separate samples of different classes, while cluster samples of the same class were remained as closely as possible at the projection point. It aimed to identify which samples enabled the minimum objective function:

1 $$J(w)=\frac{{w}^{T}{S}_{B}w}{{w}^{T}{S}_{W}w}$$

In which:

2 $${S}_{B}=({m}_{2}-{m}_{1}){({m}_{2}-{m}_{1})}^{T}$$

3 $${S}_{W}=\sum _{n\in {\complement }_{1}}({x}_{n}-{m}_{1}){({x}_{n}-{m}_{1})}^{T}+\sum _{n\in {\complement }_{2}}({x}_{n}-{m}_{2}){({x}_{n}-{m}_{2})}^{T}$$

referred to between-cluster variance and variance sum within clusters, respectively.

During classification, new samples were projected with ***w*** and the location of the projection point determined the class of the new samples.

### Construction of LS-SVM discrimination model

SVM classification was essentially defined as the maximum margin linear classifier in the feature space, aiming to identify a hyperplane in the *p*-dimensional space and to separate two classes of samples as correctly as possible. LS-SVM was a simplified and improved version of SVM that preserved the capability of processing small sample sizes, high dimension and nonlinear analysis. The improvement was represented by two factors: (1) inequality constraints in SVM were revised to equality constraints and (2) loss function was revised to squares-error loss function.

The objective function defined in LS-SVM was:

4 $${}_{\omega ,b,e}{}^{min}J_{p}(\omega ,\,e)=\frac{1}{2}{\omega }^{2}\omega +\gamma \frac{1}{2}\mathop{\sum }\limits_{i=1}^{N}{e}_{i}^{2}$$

5 $${\rm{s}}{\rm{.t}}.:{y}_{i}[{\omega }^{T}\phi ({x}_{i})+b]=1-{e}_{i},\,,i=1,\ldots ,\,N$$

LS-SVM was used to map vector **X** with regard to the primitive space. Therefore, the weight coefficient ***w*** could not be solved directly. However, the solution of the objective equation of LS-SVM could be achieved by constructing Lagrange function of the objective equation and converting to its dual problem. New samples were classified by the equation below:

6 $$y(x)=sign[\mathop{\sum }\limits_{i=1}^{N}{\alpha }_{i}{y}_{i}K(x,{x}_{i})+b]$$

### Construction of PLS-DA discrimination model

PLS-DA was essentially a special form of PLS regression. It defined dummy variables of classes as Y according to a certain rule, thereby enabling classification by regression. In general, PLS-DA merely solved binary classification problems, although the PLS algorithm could process multivariate dependent variable Y. Therefore, it could be employed for the classification of multiple classes.

### Construction of PCA-DA discrimination model

PCA-DA referred to the PCA-based DA. Therefore, with the exception of the PC number optimization, the other elements of the model were the same with those of DA.

### Multi-class classification

In the classification toolbox 5.0, multi-class classification was implemented by expanding class variables into matrices, including DA, PLS-DA, and PCA-DA, as described previously. Since LS-SVM could merely solve binary classification problems, the realization of multi-class classification also relied on decomposing the raw multi-class classification problems into multiple binary classifications, of which the results were further processed, thus achieving the classification for new samples.
